# G-CSF-Associated Bone Marrow Necrosis in AML after Induction Chemotherapy

**DOI:** 10.1155/2012/314278

**Published:** 2012-06-18

**Authors:** Ikenna Osuorji, Lyle Goldman

**Affiliations:** Department of Hematology and Oncology, Providence Cancer Institute, 22301 Foster Winter Drive, Southfield, MI 48075, USA

## Abstract

Bone marrow necrosis (BMN) is defined as necrosis of the myeloid tissues and stroma without involvement of the cortical bone. We report a case of 66-year-old male with AML-M4 (FAB classification) who was given induction chemotherapy with cytarabine and daunorubicin. Filgrastim at 480 micrograms was administered on days 15–19 to shorten the duration of neutropenia. Consequently patient developed severe pelvic bone pain, leukoerythroblastosis, and severe leukocytosis. Repeat bone marrow aspiration and biopsy on day 21 confirmed bone marrow necrosis. These manifestations responded quickly to discontinuation of filgrastim. Subsequently, he recovered full myelopoiesis. We suggest that there may be more cases of BMN associated with G-CSF that are undiagnosed.

## 1. Introduction

Bone marrow necrosis (BMN) is a rare entity [1]. It is defined as necrosis of the myeloid tissues and stroma without involvement of the cortical bone [2–4]. This differs from avascular necrosis of bone where the cortical elements are usually involved with sparing of the myeloid elements such as in sickle cell disease (SCD). However BMN has been reported in SCD in association with vasoocclusive crisis [5]. Filgrastim, a granulocyte colony stimulation factor (G-CSF), is recommended to shorten duration of febrile neutropenia and prophylaxis of neutropenia following myelosupressive chemotherapy regimen [6]. Katayama et al. reported a case of BMN in a patient with acute myeloid leukemia during administration of G-CSF in combination with conditioning regimen, for bone marrow allotransplant [7].

We report a 66-year-old male with acute myeloid leukemia who developed BMN after successful induction chemotherapy following administration of G-CSF, intended to shorten duration of neutropenia.

## 2. Case

**Figure 1:**
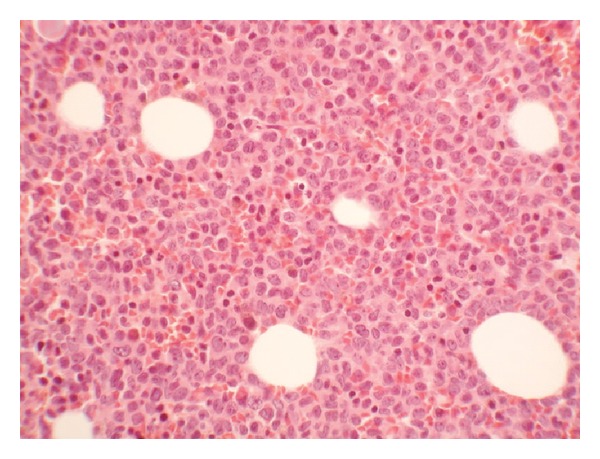
Photomicrograph of the preinduction chemotherapy of bone marrow showing diffuse infiltration by malignant cells.

A 66 year old African American male diagnosed with AML (M4, FAB classification).He presented to the hospital with leukocytosis of 26.7 K/mcL, hemoglobin of 12.4 gm/dL and platelet of 155 K/mcL with 24% blast in the peripheral smear. He had splenomegaly on examination, chest x-ray was normal and cultures were negative. Flow cytometry of initial bone marrow biopsy (Figure 1) showed 27% myeloblast, positive for CD34, CD 117, HLA-DR and CD 33 and negative for CD14, and CD56 as well as 61% monoblast positive for CD14, CD11b,HLA-DR and CD64 and negative for CD34,CD 56 and CD117. Bone marrow aspirate smear showed myeloblast, monoblast and promonocytes all together constituting 70% of cells. Cytogenetic analysis showed normal male karyotype.

He received induction chemotherapy with cytarabine at 200 mg/m^2^ days 1–7, daunorubicin 90 mg/m^2^ days 2–4, rasburicase, hydration with 5% dextrose and sodium bicarbonate infusion at 125 mls/hr.

Day 14 bone marrow aspiration and biopsy showed no residual leukemia with hypocellular marrow (10–15% cellularity), 4% myeloblast and 3% monocytes by flow cytometry.Thereafter, absolute neutrophil count (ANC) remained < 40/mcL for several days. Consequently patient developed fever and was treated with Voriconazole, Cefepime, and Vancomycin.In the bid to shorten the duration of neutropenia, Filgrastim at 480 microgram was administered for 5 days on days 15–19. While on Filgrastim he developed severe bone pain mainly in the pelvic bones which promptly resolved on discontinuation and ANC was above 5 K/mcL. However white blood count continued on the upward trend to a peak of 74 K/mcL. Peripheral blood showed neutrophilia, monocytosis and leukoerythroblastic pattern, anemia and reticulocytopenia.

**Figure 2:**
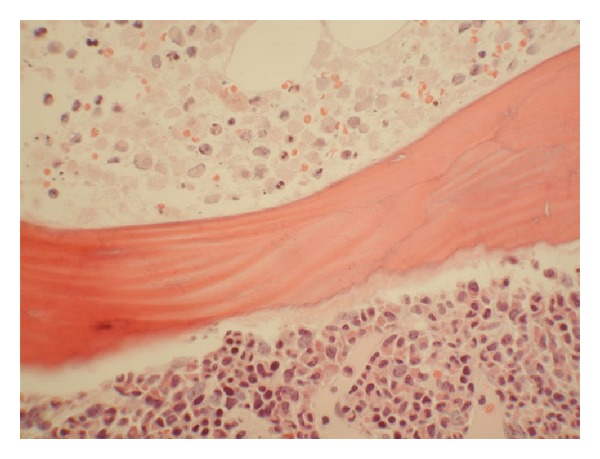
Low power photomicrograph of bone marrow showing areas of necrosis and nonnecrotic areas after G-CSF administration.

Given mounting concern for identification of definitive etiology of profound leuckocytosis, a repeat bone marrow biopsy was done on day 21, which revealed hypercellular marrow with patchy myelonecrosis and 1% myeoblast 6.8% monocytes (Figure 2). White blood cell count after the peak of 74 K/mcl started a downward trend to 15 K/mcl on discharge. LDH was 945 units/L and alkaline phosphatase was 236 units/L. Hospital course was also complicated by acute renal failure suspected to be multifactorial in etiology with peak creatinine value of 4 mg/dL and 2 mg/dL on discharge.

The patient did complete the consolidation phase of his treatment with high dose cytarabine. He received pegfilgrastim during consolidation without recurrence of BMN. He has no evidence of relapse two and half years from diagnosis.

## 3. Discussion

**Figure 3:**
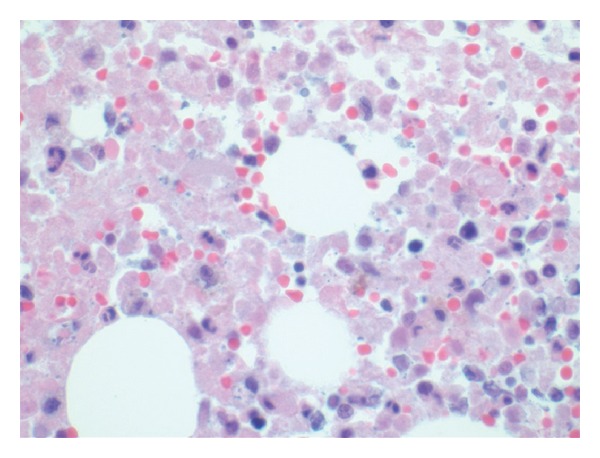
High power photomicrograph of bone marrow showing areas of necrosis and nonnecrotic areas after G-CSF administration.

Administration of G-CSF is routinely used in the recovery phase of the marrow after induction chemotherapy for AML and other myeloid malignancies [6]. To our knowledge, there is no report of BMN after achieving target hypoplasia with a cellularity of less than 5% blast cell count. However there are reports of marrow necrosis after administration of G-CSF prior to conditioning regimen and recovery phase of bone marrow following chemotherapy for non-Hodgkin lymphoma [7, 8]. It is interesting to note that our case and the one reported by Katayama et al. are both related to AML with some form of monocytic differentiation; however there is insufficient data to establish a predilection of these group of patients to the effect of G-CSF. Following induction, this patient achieved marrow hypoplasia of less than 5% blast cells in the marrow without any evidence of BMN at the 14-day aspirate and biopsy. BMN is quite evident in the 21-day sample after G-CSF administration (Figures 2 and 3). We theorizes that rapid proliferation of myeloid cell lines induced by the G-CSF led to microvascular occlusion [9] and consequent BMN. Clinical features reportedly associated with BMN include bone pain, fever, anemia, thrombocytopenia, leuckocytosis, and leukoerythroblastic differential leukopenia [1]. Of these, our patient had bone pain, leucocytosis and fever, during bone marrow recovery from induction chemotherapy.

Reported disease associations with BMN include; malignancy, infections, drugs, sickle cell disease, hyperparathyroidism, anorexia nervosa, hemolytic uremic syndrome, antiphospholipid antibody syndrome, disseminated intravascular coagulation, and also idiopathic [1]. BMN is most closely linked to malignancy (90%) and 60% linked to hematologic malignancies [1]. It is important to note that among these reports related to malignancy, BMN was mostly present at diagnosis [10–15]. This is in contrast to the iatrogenic forms.There appears to be a nonspecific association with LDH and alkaline phosphatase elevation which may be more in keeping with the primary disorder [1, 7, 8]. Of note is that both of these were elevated in our patient.

Treatment is usually that of the underlying illness [1, 7, 12, 14]. Prognosis appears to be largely dependent on the primary associated disorder [1, 11, 15].

## 4. Conclusion

BMN is rare and cases related to G-CSF administration are much more uncommon. Only one case has been previously reported. More case reports should be encouraged. Given the incidence of bone pain which occurs in 78% in patients with G-CSF administration, BMN may be more common than observed.
